# Florence Nightingale and Her “Notes on Nursing”

**Published:** 1860-05

**Authors:** 


*Florence Nightingale and her "Notes on Nursing."*—The *Medical Times and Gazette* makes the following remarks in regard to this eminent lady and her recently published book:

Nursing the sick has been with her a labor of love; the whole tenor of her writings tends to ennoble that vocation. and to redeem it from the hands of the ignorant, the stupid, and the thoughtless. With noble and most devoted energy she has always endeavored to elevate the calling of the nurse, by bringing thought, intelligence, and study to bear upon her work, and by calling forth the finer feelings of the mind in the exercise of the most humane of all vocations. It is now more than fourteen years since Florence Nightingale began to give her undivided attention to this field of thought and action. Twice has she been in training as a nurse at the Institution of Protestant Deaconesses at Kaiserwerth, on the Rhine. She has studied with the "Sœurs de Charite," in the Hospitals of Paris. She has visited the Hospitals of Berlin, and those of many other towns in Germany, She has visited those of Lyons, Rome, Alexandria. Constantinople, Brussels, and likewise the Hospitals in the chief towns of our own country; but the most extensive sphere of her usefulness, and one where her experience was most matured, was in our Military Hospitals at Scutari and the Crimea, during the Russian war. Thither she was sent by Mr. Sidney Herbert, then and now Secretary at War, who has the honor and merit of having been the first to appreciate, and to put in a position of public usefulness, the singular abilities of Florence Nightingale. What she succeeded in doing at Scutari and elsewhere for our sick and wounded soldiers is now a matter of history. What she has since done in bringing about sanitary improvements in our own army has still to be recorded. She has never been at rest since her arrival in this country. With such precedents and with such extensive experience, acquired among scenes of most varied suffering, can any one doubt that a written record of her thoughts and ideas regarding the subject of nursing the sick can be other than of the greatest possible public interest? She has undoubtedly ennobled the calling of the nurse, she has made her vocation a labor of love, and has sacrificed her health in the acquisition of her extensive experience. She has brought to bear upon the subject all the energies of an active and highly cultivated intellect, rendered still more energetic by intense enthusiasm in the work. The asperities of business not unfrequently encountered in the rough walks of life through which she has passed, have been at once smoothed down, or have altogether disappeared through the influence of that remarkable tact with which she is so remarkably gifted, directed by a mind the most amiable, gentle and refined. We think, therefore, we are justified in the belief we have expressed, that no other living person than Miss Nightingale, could write a book on nursing such as we have now before us. Every line of it, from the preface to the end, rivets the attention, every paragraph is suggestive, every page carries the reader into a world of thought.—*Medical and Surgical Reporter.*

